# Perception and experiences of adolescent mothers and communities in caring for their preterm babies: findings from an in-depth study in rural Bangladesh

**DOI:** 10.1186/s12884-024-06345-x

**Published:** 2024-02-17

**Authors:** Shumona Sharmin Salam, Ahmed Ehsanur Rahman, Shema Mhajabin, Tapas Mazumder, Tamanna Majid, Md. Taqbir Us Samad Talha, Rajib Haider, Anika Tasneem Chowdhury, Sharmin Islam, Shafiqul Ameen, Sabrina Jabeen, Julie Balen, Shams El Arifeen, Quamrun Nahar, Dilly OC Anumba

**Affiliations:** 1 Department of Oncology and Metabolism, University of Sheffield, Sheffield, UK; 2 International Centre for Diarrhoeal Disease Research, Bangladesh (icddr,b), Dhaka, Bangladesh; 3 Health Research Institute, University of Canberra, Canberra, Australia; 4 School of Health and Related Research (ScHARR), University of Sheffield, Sheffield, UK; 5 School of Allied and Public Health Professions, Canterbury Christ Church University, Kent, UK

**Keywords:** Adolescents, Preterm birth, Bangladesh, Maternal, Newborn

## Abstract

**Background:**

A significant concern for Bangladesh is the high prevalence of adolescent pregnancy and the associated negative consequences for mother and baby, including a teen-related increased risk of preterm birth (PTB). Bangladesh also has one of the highest incidences of PTB (19%). Despite these high numbers of adolescent pregnancies and PTB, little is reported about the experiences of adolescent mothers in caring for their preterm babies, and the interventions needed to support them. The aim of this study was to explore gaps and opportunities for improved care for preterm babies among adolescent mothers and communities in rural Bangladesh.

**Methods:**

We conducted a qualitative study in rural villages of Baliakandi sub-district of Bangladesh. Data collection involved in-depth interviews with adolescent mothers of premature and term babies, adult mothers with premature babies, and family members (*n* = 36); focus groups with community members (*n* = 5); and key informant interviews with healthcare providers (*n* = 13). Adolescent mothers with term and adult mothers with PTBs were included to elicit similarities and differences in understanding and care practices of PTB. A thematic approach was used for data analysis.

**Results:**

We explored two major themes- perceptions and understanding of PTB; care practices and care-seeking for illnesses. We observed gaps and variations in understanding of preterm birth (length of gestation, appearance, causes, problems faced) and care practices (thermal management, feeding, weight monitoring) among all, but particularly among adolescents. Immediate natal and marital-kins were prominent in the narratives of adolescents as sources of informational and instrumental support. The use of multiple providers and delays in care-seeking from trained providers for sick preterm babies was noted, often modulated by the perception of severity of illness, cost, convenience, and quality of services. Health systems challenges included lack of equipment and trained staff in facilities to provide special care to preterm babies.

**Conclusion:**

A combination of factors including local knowledge, socio-cultural practices and health systems challenges influenced knowledge of, and care for, preterm babies among adolescent and adult mothers. Strategies to improve birth outcomes will require increased awareness among adolescents, women, and families about PTB and improvement in quality of PTB services at health facilities.

## Background

Adolescent pregnancy and motherhood continues to be considered a priority public health and human rights issue because of its profound consequences and long-term negative effects on young individuals, their families, and entire communities. Globally, 16 million adolescent mothers aged 15–19 years and 2 million below the age of 15 experience the physically and emotionally demanding journey of pregnancy and childbirth every year [1, 2]. More than 90% of these births occur in low- and middle-income countries (LMICs) and within marginalized communities – commonly driven by poverty, lack of education and employment opportunities in these areas [3–5]. Adolescent childbearing is generally associated with negative health consequences for both the mother and baby including increased risks of maternal and neonatal deaths, stillbirths, preterm births (PTB), small-for gestational-age (SGA) babies, severe neonatal complications, pregnancy and childbirth complications, and maternal undernutrition, as compared to child-bearing for those older than 19 years [6–10]. In addition, adolescent mothers must simultaneously adapt to the demanding role of being a mother and nurturing a baby while they are still going through their own biological, physical, emotional, and psychological development as an adolescent [11–13]. Without the necessary knowledge, skills, and resources to deal with early parenthood, they face several social, economic, personal and relational challenges [11–13].

Both hospital and population-based studies in high and low resource settings consistently report that adolescent mothers are at an increased risk for PTB compared with mothers aged above 19 years of age [6, 7, 9, 14–16]. Multi-country studies conducted in LMICs also identified that adolescent mothers aged less than 20 years are at increased risk of PTB, after controlling for country, health facility effects and for potential confounding factors [7, 10]. The risk of PTB is especially high among adolescent mothers less than 16 years of age [7, 10]. Although all babies are vulnerable in the first few days after birth and require essential newborn care, premature babies are especially vulnerable to temperature instability, feeding difficulties, low blood sugar, infections, and breathing difficulties [17]. Since preterm babies are high-risk neonates requiring special attention and care, early recognition and health care-seeking is very important for these mothers and babies, to reduce morbidity and mortality. However, this extra care might be challenging for adolescent mothers. Thus, already higher risks posed to adolescent motherhood may be further compounded by a PTB, and vice versa, elevating the vulnerability and distress among adolescent mothers and their high-risk babies.

Despite remarkable progress in health indicators, a significant concern for Bangladesh is the high prevalence of child marriages and the subsequent high levels of adolescent pregnancy. According to the recent 2017-18 Bangladesh Demographic and Health Survey (BDHS) the median age at first marriage among women aged 20–49 is 16.3 years and approximately 71% of women in this age group were married by age 18. The report also highlights that almost one-third (28%) of adolescent girls aged 15–19 years have begun child bearing [18]. Moreover, Bangladesh has one of the highest incidences of PTB in the world. An estimated 603,698 babies were born prematurely in 2014 i.e., 19% of the total number of births that year [19]. The BDHS estimates that about 19% of all neonatal deaths are directly attributed to PTB [18]. Another study in rural Bangladesh found the risk of death in preterm babies to be significantly higher than that of term babies [20]. Given that worldwide, almost half of preterm babies are born at home and that even among those born in facilities critical newborn care is often lacking [17], ensuring that adolescent mothers, their families and communities are well-informed and empowered about care of preterm babies is crucial [17].

In spite of the high rate of adolescent pregnancies and preterm births in Bangladesh, there is a dearth of evidence documenting the understanding and experiences of adolescent mothers in caring for their preterm babies. Equally, little is known about the interventions needed to prepare and assist them in caring for their premature babies. Exploratory studies in African settings, including Malawi, Uganda and Ghana, indicate that inadequate care of preterm babies is commonly driven by a (i) lack or inadequate knowledge about the causes of preterm birth, diseases severity and how to care for preterm babies; poverty which prevented families from buying warm materials, living in properly build warm houses or paying transport costs and costs of health facilities; and (iii) poor quality of care at health facilities due to lack of protocols, skilled service providers and basic equipment, drugs and other supplies [21–24]. In addition, findings from these studies as well as in Bangladesh reveal low rates of care-seeking or using traditional medicines and care-seeking from unqualified providers for complications and illnesses in preterm babies [21–25]. However, whether these factors are important barriers in other settings specially among adolescent mothers in rural Bangladesh is unknown. We, therefore, undertook a qualitative study to explore the perception and caring practices of adolescent mothers, their family and community members regarding PTB, at a rural sub-district in Bangladesh. To gain an in-depth understanding of whether and how the perception and care-practices differ between adolescents with premature births and their term counterparts, data was also collected from adolescent mothers (15–19 years) who gave birth to term babies and older mothers (≥ 19 years of age) who gave birth to premature babies. Information on the perceptions of PTB, access to related health services and subsequent management practices among adolescent mothers and community, will be important for reproductive health programs to better understand their specific needs and to tailor services and strategies to improve perinatal and newborn care in Bangladesh and other resource poor settings.

## Methods

### Study design

We adopted a qualitative approach involving in-depth interviews (IDI), key informant interviews (KII), and focus group discussions (FGD) to generate a detailed understanding of mothers’ and community perspectives regarding PTB and their experiences in caring for a preterm baby within their social context.

### Study setting and participants

The study was conducted in rural villages of Baliakandi sub-district in Rajbari District, central Bangladesh. The sub-district was chosen as International Centre for Diarrhoeal Disease Research, Bangladesh (icddr,b) operates a demographic and health surveillance system among a population of approximately 200,000 in 261 villages in the sub-district. The surveillance system collects information on key demographic and reproductive events every four months including age, births, gestational age at births etc. which facilitated identification of participants for this study. The primary participants for this study were adolescent mothers aged 15–19 years who had given birth to preterm babies (any live birth before 37 completed weeks of gestation) in the 6 months prior to the start of data collection. However, we also aimed to explore whether perception, experiences and care-practices varied by age of mother and gestation of baby i.e., whether perception or experiences of adolescent mothers with a PTB differ or is similar to adolescent mothers with term birth or adult mothers with PTB. As such, data was also collected from adolescent mothers (15–19 years) who gave birth to a term baby and older mothers (> 19 years of age) who gave birth to a preterm baby in the six months prior to the start of data collection. The secondary participants of the study included immediate family members (e.g., fathers and grandmothers of the preterm baby), community members (other mothers, elderly women and fathers) and health care providers. Immediate family members were included to obtain detailed understandings of their experiences surrounding the birth and care of the baby. To understand and obtain more information about cultural norms and social practices regarding PTB in the community, FGDs were conducted with several homogenous groups including young mothers (with preterm or term births), elderly women and fathers. In addition, we conducted key-informant interviews (KIIs) with health care providers selected purposively from public and private health facilities providing newborn and child care in the area to understand the challenges faced in providing services to preterm babies.

### Data collection

Data was collected by trained social science researchers and anthropologists between July 2019 and December 2019. Individual IDIs, FGDs and KIIs were conducted to add breadth to the data and triangulate the findings (Table 1). Interview guides were developed, translated into the local language Bangla and pilot tested. All questions were open-ended with prompts to elicit details and description from the participants. Topics covered in the interview guides included understanding of PTB, experiences with pregnancy and delivery, care practices of preterm babies, care-seeking for illnesses and the associated challenges. We conducted 36 IDIs; including adolescent mothers with preterm or term babies (*n* = 18), adult mothers with preterm babies (*n* = 10), family members (*n* = 8). We conducted KIIs with formal and informal health care providers (*n* = 12) (Table 1). This included nurses in labour and neonatal wards (*n* = 3), obstetricians and gynaecologists (*n* = 2), neonatal consultant (*n* = 1), health care worker (*n* = 1) and traditional birth attendants (*n* = 3). FGDs were conducted with young mothers (*n* = 1), grandmothers (*n* = 1), fathers (*n* = 2), village doctors/community people (*n* = 1) (Table 1). There was no overlap in participants of IDIs, FGDs or KIIs. Each interview/discussion was conducted in a private location at a time that was convenient to the participant or group. Prior to starting the interviews or FGDs, written informed consent or assent was administered. Written informed consent was obtained from study participants above 18 years of age. For participants less than 18 years of age, assent from them and informed consent from their legal guardian was obtained. Each FGD had six–eight participants and efforts were directed to keep the groups as homogenous as possible. Each of the interviews and focus groups lasted between 30 and 115 min and, with the permission of the participant or group, the entire discussion was audio-recorded while a note-taker additionally took handwritten notes. Immediately after each interview or FGD, a short summary was prepared, noting any important points or challenges to facilitate with data analysis. Data collection continued until saturation was reached.

**Table 1:** Data collection methods

Interview type	Number of participants (*n* = 73)
IDIs	
Adolescent women with PTBs	10
Adolescent women with term births	8
Adult women with PTBs	10
Grandmothers	3
Fathers	5
*Sub-total*	***36***
KIIs	
Birth attendant	3
Trained/Formal health care provider	9
*Sub-total*	***12***
FGDs	
Elderly women	2 groups (12)
Fathers	2 groups (16)
Young mothers	1 group (6)
*Sub-total*	***5 groups (34)***

### Data analysis

Preliminary data from this qualitative exploration has been analyzed using an inductive thematic approach and both data collection and data analysis were conducted simultaneously [26]. Audio recordings of all FGDs and interviews were transcribed verbatim in Bangla, by the respective interviewer immediately after the interview, preventing minimal loss of data. By adding any additional notes (field notes) transcripts were expanded. Transcripts were randomly checked against audio recordings to ensure quality of transcription. An iterative process was used to code and develop themes. To begin with, initial transcripts were read and re-read carefully to familiarise with the data and a coding structure created. Following this, the important and most frequent codes were applied to the new data. Simultaneously, new and emerging codes were added to the coding structure. Finally, codes were grouped together to develop categories and collated into potential themes and sub-categories. Data analysis was carried out using NVivo v 12 Analysis Software.

## Results

In this paper we have presented perceptions and experiences of adolescent mothers on PTB, comparing it with the views of adult mothers, family and community members. Information on challenges of caring for PTB at health facilities were obtained from heath care providers. We concentrate on the two major themes emerging from our data: firstly, perceptions and understanding of PTB (length of gestation, causes, appearances); secondly, care practices and care-seeking for preterm babies.

### Background characteristics

We had a mix of participants by birthplace (facility or home birth), delivery mode (normal or c-section births), and family type (nuclear or extended) in both groups - adolescent (15–19 years) and adult (20–36 years) women with recent births and their family members (Table 2). However, a majority had six or more years of education, were housewives living with extended families, delivered at health facilities, and had normal vaginal deliveries. The mean gestational age for PTBs was 31 weeks (28–36 weeks). The fathers who participated in the IDIs were between 19 and 35 years (mean 25.2 years old), grandmothers were between 41 and 50 years (mean 46.7) years, whereas the key informants were between 30 and 86 years (mean 51.6 years) old.

**Table 2:** Background characteristics of women with recent preterm/term births

	Adolescents with PTB	Adults with PTB	Adolescents with term birth
	*n* = 10	*n* = 10	*n* = 8
**Education**			
No/Primary (1–5 y)	4	3	1
Secondary + (6 + y)	6	7	7
**Religion**			
Islam	9	9	7
Others (Hindu)	1	1	1
**Child status**			
Alive	8	7	8
Dead	2	3	-
**Occupation**			
Housewife	10	9	2
Student	-	1	-
**Family type**			
Nuclear	2	5	2
Extended	8	5	6
**Delivery place**			
Home	3	4	4
Facility	7	6	4
**Delivery type**			
NVD	8	5	5
C/S	2	5	3
**Birth Order**			
Primiarous	7	1	8
Multiparous	3	9	-
**Mean gestational age at birth (weeks)**	30.9	31.0	39.4

### Perception and understanding of PTB

#### Length of gestation

The overall concept of babies born preterm was well understood among all mothers and community members in Baliakandi. Locally, PTBs were referred to as *“births that occur before time”* or “*shomoyer age”* and this appropriate time was mostly defined as the time needed for the baby in the womb to become *fully or well-nourished* or “*pushto.*” The length of gestation was always described in months rather than in weeks and we observed variations in participants’ opinion as to what that appropriate time or length of gestation should be. The most common responses by adolescents with PTBs included, nine months, nine months 10 days, between nine to 10 months, 10 months, 10 months 10 days whereas other adolescent mothers with term births or adult mothers with PTBs mostly mentioned 10 months. Any births at the 8th or 7th month were considered by all to be early.

*“Elders say that a baby is healthy if born at 10 months 10 days and doesn’t have problems with being undernourished.” (IDI03 Adolescent mother with a preterm baby)*.

*“When the baby is nourished… if the baby stays in the womb for nine months, then it is nourished.” (IDI27 Adolescent mother with a preterm baby)*.

The responses of family members and other community participants varied like those of adolescent mothers with premature births, with some mentioning that although 10 months is ideal, nine months is also good: *“Baby is nourished if 10 months… but nine months is also not bad, its medium…” (FGD01 Elderly women)*.

All participants used or were aware of using the date of last menstrual period (LMP) to estimate the month of pregnancy and birth. However, participants also relied heavily on dates provided during ultrasonography (USG) to estimate their due dates. Often these USGs were done during the 2nd or 3rd trimester to know the status of the baby or to estimate the gestational age in cases where mothers were confused with their LMP date, for example those with irregular periods or spotting during pregnancy, as one participant mentioned:

*“I couldn’t remember clearly when I became pregnant. So, I went to do an ulta (USG) to determine the date…I have heard that you can even tell how many months old the baby is if you do an ulta (USG). I told them I forgot the date of my last menstruation. Accordingly, they gave me a date of delivery.” (IDI11 Adult mother of a preterm baby)*.

#### Appearance of PTB

When mothers and community members were asked to describe a preterm baby, they used several external physical features. The most common notion mentioned by all participants was that babies born before time are under-nourished (*opusto*), small (*choto*), weak (*kabu*), have less weight, frail (*finfina*), have long and thin hands, and legs *(haat pa. noli noli*). The term *opusto*, which literally means being under-nourished, was mentioned by all participants to describe preterm babies and was often synonymously used to mean babies who are not fully grown. For babies born as early as seven months, a common way to identify them, as mentioned by adolescents as well adults, was that the baby’s eyelids are fused together (*chokh fute na*). Other terminologies used by older participants and community members who had seen preterm babies included visible veins around the stomach (*pet-er rog dekha jai*), wrinkled skin, sunken forehead, and no hair.

#### Problems preterm babies may face

Participants highlighted problems that preterm babies may suffer from, or their preterm babies had suffered. Adolescent and adult mothers with preterm babies, recollected problems mainly from their experience, whilst about half of the adolescent mothers with term babies mentioned that they were not aware. Participants of FGDs, family members and adult mothers, however, could promptly mention problems faced by preterm babies. Several participants including adolescent mothers emphasized that babies born at nine months or later are generally healthy whereas babies born at seven months rarely survive. A mother mentioned, *“babies who are born in the 7th or 8th months are not born with good health. Those who are well-nourished, survive and those who aren’t, die” (IDI14 Adolescent mother of a preterm baby)*, whilst others highlighted the need to spend a lot of money to ensure survival, or shared recollections of incidents where babies born early were kept in *glass houses* (*Kacher ghor*- incubators), some of whom had survived:

*“…babies born at seven months may not survive and those at 8 months have more possibility but need to spend a lot of money…” (IDI12 Adult mother of a preterm baby)*.

*“The baby was not nourished as much as he would have been if born after 10 months. His parents took him to hospital where the baby was kept in a glass house [incubator] so that his eyes develop. They returned after three months when the baby started feeding.” (FGD04 A participant of FGD with fathers)*.

Other problems mentioned include babies are undernourished; eyelids are fused, babies have difficulty breastfeeding; have breathing difficulties requiring hospitalization; baby turns blue or black; more prone to diseases (such as cough and cold, diarrhea, pneumonia etc.) due to the babies being undernourished; physically and mentally disabled; ongoing poor nutritional state, and that their health doesn’t fully recover when they grow up.

*“Baby may suffer from heart problem, or be weak or their eyes may not develop.” (IDI27 Adolescent mother of a preterm baby)*.

*“Baby will either be less intelligent or disabled. Or he will have any other parts of his body damaged…he may have underdeveloped eyes, or hands or legs…he may not be talented. He did not complete his full growth.” (FGD04 A participant of FGD with fathers)*.

*“After delivery, the two big problems are difficulty in breathing and cough. Makes wheezing sound inside the throat, noses are blocked, and baby cannot breathe. These happen more to babies born in the 7th or 8th month.” (KII06 Untrained birth attendant)*.

#### Causes of PTB

Findings revealed that awareness about the causes of PTB is limited in rural Bangladeshi settings and that many of the perceived causes are outside modern medicine. Over half of the adolescent mothers (10 out of 18) and half of the adult mothers (five out of 10) could not explain why babies could be born prematurely or why her newborn was born premature. Other participants, either mothers or family and community members, mentioned several causes which have been grouped into (i) maternal health related, (ii) lifestyle or behavioral, and (iii) supernatural/spiritual causes (Table 3).

**Table 3:** Perceived causes for PTB

**Maternal health**
Less maternal age / less maternal age leading to c-section
Poor nutritional status
Use of medications (fever, deworming etc.)
Inadequate sleep
Falls
Trauma to abdomen
Excessive vaginal discharge
Low-lying placenta
Convulsions, eclampsia
Urine infection
Indicated c-section Sex during pregnancy
**Lifestyle and behavioural**
Heavy work, lifting heavy objects (using tube-well etc.)
Inadequate eating
Not taking recommended vitamins, calcium, folic acid during pregnancy
Being beaten by husband
Stress due to familial issues e.g., disagreement with in-laws, husband’s extra marital affairs
Attempted abortion
Elective c-section
**Supernatural/spiritual**
Evil spirits (being possessed by Jinn, *dosh, chut, upri, groher* *shomossa, bhoot)*
God’s will

Poor nutritional status of the mother or inadequate eating was one of the most mentioned causes for premature birth. Because premature babies were born small and weak, many participants, including mothers, opined that this was because the mother was weak, under-nourished, or because she did not eat enough food or vegetables or take the recommended vitamins, iron, or calcium medications:” My *baby was born early, as I didn’t get enough to eat…” (IDI08 Adolescent mother of a preterm baby).* On the contrary, one young mother mentioned that food or eating habits had no effect on premature birth, as her relative who had a similar outcome used to eat adequately, whilst another mother stated that in order to prevent PTB and unnecessary c-sections, pregnant women should eat smaller but frequent meals, so that the baby does not grow too big in the womb.

Trauma to the abdomen, accidents/falls, risky work, lifting heavy objects were also mentioned by a few participants across the different groups. According to a young man whose child was born premature: *“We are farmers, we go to the field. Then there is rice to look after in the house or five cows. I would ask her to put the rice away. There is always some work at home, she did a lot of risky work…she used to carry heavy items and that is why my child was like this (born preterm)” (FGD04 A participant of FGD with fathers).*

Use of medicines during pregnancy such as for fever, pain or deworming was highlighted during FGDs. A participant of an FGD highlighted that, failed abortions using abortive medications may later result in early delivery or even still-births. Another mother whose twin preterm babies had died, expressed concern and confusion whether taking iron-folic acid and calcium medications had resulted in such an outcome or not.

Many mothers and community participants believed that supernatural or spiritual causes such as God’s will or evil spirits (*dosh, chut, upri, groher shomossa, bhoot, Jinn*) were to blame. The opinions related to evil spirits were divided, and some young mothers and husbands strongly opposed the notion, mentioning that these were myths and old tales, whilst others said this might result in miscarriage or inability to get pregnant but not premature births. Those who believed in the notion mentioned that pregnant women are to follow certain norms during their pregnancy e.g., not leaving the house in the evening, afternoon, prayer times, or not roaming in the garden with big trees. It was said that an inability of the pregnant women to do so may result in early birth or even loss of pregnancy, as the excerpt below reveals:

*“…Women are prone to curses and evil spirits, for this reason some babies get aborted at eight months. These babies may be born alive for some women but for others they die before birth…for example today is full moon, a lot of things (sprits) are outside during this time…if I am a new pregnant woman (kacha poyati), the evil spirits can attract me… when it attracts a pregnant woman, the pregnant women loses appetite, becomes thin. Sometimes she starts having abdominal pains. That is why she delivers early or loses her pregnancy.” (KII06 Untrained birth attendant)*.

An adult woman with a PTB strongly believed that she was possessed by *Jinn. In her words*: *“While I was sleeping, I used to feel that there’s someone else sleeping beside me…the kabiraj [traditional healer] said that a huge Jinn is after me but wasn’t courageous enough to harm… the kabiraj gave me an amulet that I wore till seven days after delivery…*” (*IDI13 Adult mother of a preterm baby*). Now she believes that her newborn is possessed by the *Jinn* as the baby cries a lot and turns black. Similarly, two participants with prior history of PTBs believed that their womb was cursed, and another woman with two PTBs and whose husband’s second wife had seven miscarriages believed that someone had cast an evil eye on their house.

Unable to explain the cause of PTB, some participants opined that it was God’s will. A husband mentioned that: “*I don’t know the reason. Life and death depend on The One. We don’t have the ability to know when one will be born or die!” (IDI36 with a Husband).* Another grief-stricken young mother said, *“People used to ask me, what I have done that the baby was born at seven months. But I did not do anything…God took away whatever was His in the way He wanted…” (IDI07 Adolescent mother of a preterm baby).*

A few husbands/fathers during in-depth interviews as well as in the focus groups mentioned that a lower maternal age to be a cause of PTB as well as other complications for the mother and child, including miscarriage and early c-sections. According to these men, young mothers are usually poorly nourished which makes it difficult for them to bear a child, as one husband stated, *“girls who deliver early get pregnant at young age…nowadays girls are married at 12 or 13 years of age…if someone is married at a young age, they are not fully nourished, how will she bear a fully nourished child (FGD06 A participant of FGD with fathers).”* Unexpectedly, the two traditional birth attendants interviewed strongly believed that it is easier for adolescent mothers to deliver since their bone structures were believed to be very flexible and not rigid like those of older women. Other infrequently mentioned causes included infections, vaginal discharge problems, low-lying placenta, convulsions, domestic violence, and stress.

### Care of preterm babies

In our study we asked participants about the care a preterm baby would need with an aim to elicit information on knowledge and practices related to three important aspects of preterm babies’ care: temperature management, frequent feeding, and weight monitoring. In general, care practices did not vary by mothers’ age (adolescent or adult) but rather on the status of the baby.

Having identified preterm babies to be small, under-nourished and weak, both adolescent and adult mothers mentioned the need to provide extra care to preterm babies. However, it was difficult for participants – especially adolescents – to describe what this extra care should entail, especially when physical problems were not present. For example, a baby who was born a month earlier was perceived to be under-nourished but otherwise well if there were no visible signs of illnesses or problems/anomalies and cared for like term babies: *“…baby was otherwise healthy and beautiful at birth. Baby was under-nourished and hence was not so active…” (IDI03 Adolescent mother of a preterm baby).* For these babies, the routine care at home included feeding the child well, keeping them warm, clean and dry, maintaining cleanliness including timely bathing so that the baby becomes healthy or so that the baby does not fall sick. An untrained birth attendant mentioned the need for a three-way approach for care of preterm babies that included (i) extra care by mother, (ii) traditional/spiritual care for dealing with evil eyes/spirits and (iii) treatment from doctors depending on the condition of the baby:

*“If you enclose the house, then the baby will be free from evil eyes or curses – this is spiritual or traditional treatment; If the child is under-nourished, or has cough and cold or gas, you need advice from doctors. In addition, mother needs to take extra care such as timely feeding, bathing, sleeping etc. In this way the child will become healthy in a few days.” (KII06 Untrained birth attendant)*.

The recommendation for babies that were born too early, or babies born too small was that advice should be sought from formal health care providers and care provided accordingly. Although participants could not mention the benefits or purpose of using incubators, a few recollected instances where babies born early were kept in glasshouses and treated till they could be brought home.

Findings also highlight that adolescent mothers were dependent on various members in their network for support and care for the preterm/term newborn. These sources varied by the type of support needed and the roles of natal and marital kins were found to be prominent in their narratives. While the role of the adolescent’s mother in providing instrumental and emotional support was a constant theme, other members such as mothers-in-law, aunts and sisters were also important. These are highlighted in the excerpts below:

*“My mother taught me this (how to breastfeed). I did not know how to. She told me the proper way to hold when breastfeeding so that the flow of milk is slow. Otherwise, the baby will choke.” (IDI01 Adolescent mother of a term baby)*.

*“I was afraid to take care of baby, my mother used to do everything, from bathing to cleaning. Now I have learnt” (IDI03 Adolescent mother of a preterm baby)*.

#### Temperature management

In rural Baliakandi, all newborns (preterm and term) were considered to be susceptible to cold and hence need to be kept warm: *“Newborns come from the womb, they need to be kept warm.”* The threat of *“catching cold”* was more imminent among preterm babies since they are small and under-nourished. Several practices were followed to keep them warm and prevent the babies from catching a cold.

Our findings reveal that, in general, management of temperature depended on the weather or external temperature. Whilst all newborns are wrapped using a *katha* (traditional cloth made from old sarees) or towel immediately after birth, as days progressed newborns or babies were cared for according to the external temperature which included wearing warm clothes or covering the child with layers of cloth during rainy or winter days or during evenings and wearing thin clothes or using the fan during hot days. Among mothers with preterm babies particularly small or early preterms, practices included wrapping them in blankets, cotton, or holding the baby in mother’s arms (to receive mother’s warmth – *mayer om*) often at the advice of health care providers or elders. However, participants could not clearly specify how long they did this for and gradually started dressing newborns as per temperature.

Regardless of the communities’ awareness about keeping newborns warm, traditional practices of immediate bathing after birth were prevalent. An adolescent mother of a preterm baby mentioned: “*We didn’t bathe her for nine days. It is customary to bath babies after they are born, to clean all the impurities. So, on the 9th day my cousin sister-in-law shaved her hair off. We did not bath her fully but sprinkled some water over her body. That night the baby developed slight cough and cold*” *(IDI07 Adolescent mother of a preterm baby)*. The adolescent mother then explained that her baby died on the way to the hospital.

A few mothers of preterm babies who experienced facility births or births with health care worker mentioned delaying first bathing to seven or 12 days as the doctor or health worker had suggested that their baby was too small. Some mothers and grandmothers whose babies were born at health facilities expressed discontent that in the absence of any bathing facility they had to bathe the baby once they returned home. Almost all babies were massaged with mustard oil on their skin before bathing. The mustard oil was seen to act as a protective barrier between the baby and the water, insulating the baby from cold. Almost all babies were bathed with lukewarm water, around mid-day. The water was warmed either by placing the container under the hot sun or over a hot stove during cloudy days. Babies were bathed almost every day except for cloudy, rainy or cold days, when bathing would be skipped, or they would be wiped with a wet cloth.

#### Feeding

Participants in our study widely shared the view that for under-nourished and weak preterm babies to grow healthy and regain their strength they must be fed well. For extremely preterm babies, who were not able to suckle, the practice was to express milk and feed with a dropper or spoon. For other preterm babies, feeding practices varied and some participants also mentioned pre-lacteal and non-exclusive breastfeeding. Honey and warm water (“to clear the stomach”) were the two common pre-lacteal feeds mentioned. The common reason for non-exclusive breastfeeding with formula or cow or goat’s milk was that the mother was not producing enough milk. On the other hand, mothers with hospital deliveries also recollected instances where they were told off by doctors for pre-lacteal feeding or had to stop feeding babies formula milk based on the doctor’s advice. One mother mentioned, *“I started feeding after a day…the doctor scolded us a lot for feeding the baby some warm water (IDI27 Adolescent mother of a preterm baby)”* while another mother mentions, *“…we used a dropper to feed the baby powder milk (formula milk)…everyone else was feeding so we thought it would be okay… but the doctor told us that it is not good for baby, so we stopped (IDI22 Adolescent mother of a term baby).*” In terms of interval or frequency, most participants mentioned that they fed their babies when they cried while some mentioned that they fed at regular intervals.

#### Weight monitoring

Although service providers were aware of the importance of weight monitoring, none of the interviewed participants was aware of the need to regularly monitor the weight of preterm babies. Birth weight was measured only for babies born in health facilities or when care was sought from formal health care providers.

### Care-seeking for preterm babies and illnesses

Several factors influenced appropriate care-seeking for preterm babies as well as those with illnesses. These included a lack of knowledge about prematurity and perceived severity of illness, low decision-making ability and autonomy at the societal level, influence of family members and neighbors at the interpersonal level and several health systems factors such as unavailability of services and poor quality of care. These factors affected illness recognition, caused delays in decision-making regarding choice of care and uptake of appropriate services at the health facility. Adolescents were found to have low decision making ability and often consulted family members before care-seeking.

Many mothers and family members frequently mentioned that preterm newborns were small, weak, and less mobile. In the absence of any visible signs of illnesses they considered that with appropriate feeding these babies would recover. As such, care was only sought when the babies presented with illnesses. A mother who was unable to understand the severity of the child’s condition who was placed in an incubator mentions that initially they did not want to take her (now deceased) baby to a specialised hospital, despite being referred. In her words: *“My mom said baby was well, was moving and looking unlike her older grandchild. So, she did not want to take her to hospital” (IDI09 Adult mother of a preterm baby).*

Participants’ mentioned a range of health care providers for the treatment of their babies, including untrained traditional and spiritual healers, herbalists, homeopaths and village doctors. Participants claimed that they were often the first point of contact, and care was sought from qualified providers when the illness became severe or prolonged, as a father participating in a FGD highlights: *“If the baby cries a lot or has gas we go to kabiraj…For minor illnesses there is community clinic where services are free, people are poor here…then some also go to village doctors…if the baby does not recover we go to shishu hospital.”* Mothers were also often found to use amulets and spiritual water to ward off evil spirits or if the child was too cranky. Adolescent mothers also had little decision-making authority – the decision to seek care was usually made by the husband, in-laws or parents, or in consultation with them: “*I wanted to take my baby to the big hospital. But my in-laws did not approve. So, I went to the nearby doctor (village doctor) instead…” (IDI08 Adolescent mother of a preterm baby).*

Cost was another factor that was repeatedly mentioned by mothers and community members during the interviews. During the interviews, several participants highlighted that some preterm babies need specialised care or need to be kept in incubators (*glass-houses*) which were considered to be expensive. An adult mother of a preterm baby described two instances where one of them survived as the family was able to seek advanced and expensive care while the other was not: *“…see he was rich, do you understand…he took his child to Dhaka (the capital) and saved the baby by putting him inside the glass house (incubator)…whereas the other family used to live from hand-to-mouth…they waited for seven days and saw the baby was fine and stayed at home, but the baby died…” (IDI31 Adult mother of a preterm baby).* An adult mother mentioned that they were denied care at a health facility based on the assumption that they might not be able to afford the cost of the treatment whilst another adolescent mother waited for her in-laws to come and help because she had already spent a lot of money: *“The baby was very small and malnourished (pusto chilo na), but the doctor said if you take care well, she might survive. The doctor also told us to put the baby in a glass-house. However, we brought her home before we took her there. My in-laws were not at home, and we had already spent a lot of money at the hospital. So, we waited for them to come before admitting her in the glasshouse…” (IDI07 Adolescent mother of a preterm baby).*

At the health facility level, health care providers interviewed mentioned several challenges affecting service provision. For example, several service provider highlighted the lack of equipment and trained staff, as highlighted in the following excerpt: *“We are supposed to keep one baby in SCANU/warmer, sometimes I put in three babies…we have CPAP, but don’t have the disposable equipment for it…we can’t provide up to the mark service all the time…I also don’t have enough trained staff…”(KII01 -Trained service provider).* Related to this was the unavailability of a KMC ward, particularly in public hospital settings which demotivated mothers to practice KMC in front of other patient and families: *“In the absence of dedicated KMC ward, mothers don’t want to do KMC…” (KII01-Trained service provider).* In addition, another service provider highlighted the ineffectiveness of the referral system and pointed out the challenges of parents and families in arranging their own transport causing delays: *“If we cannot provide treatment, we refer…but the patient has to arrange the transport…we do not have a system…” (KII02-Trained service provider).*

Other challenges that service providers mentioned were related to availing services by the users and included late presentation or delay in care-seeking from the health facility, wanting to leave quickly from the health facility due to cost or other responsibilities and not coming back for regular follow-ups. These are highlighted in the excerpts below:

**Late presentation.***“Preterm babies are brought to the hospital very late…we receive a lot of cases…”(KII03 Trained service provider)*.

**Early discharge.***“Patients here are poor, they want to leave early…they don’t stay to see if weight of baby is steadily increasing or not…” (KII-04 Trained service provider)*.

**Lack of follow**-up. *“Some patients come for follow-up others don’t unless the baby is ill” (KII05 Trained service provider)*.

On the other hand, mothers and family members who sought care at health facilities mentioned a lack of responsive and respectful care including poor communication, denial of care, verbal abuse, and non-consented care. Among these, verbal abuse was commonly mentioned. Two mothers reported that it was difficult to reach doctors and nurses who used to get angry if the patients’ family enquired about them or asked them to come and check on their baby: *“We had to call the doctor to come…if you called them more than twice, they would get angry and scold us…” (IDI08 Adolescent mother of a preterm baby).* Other mothers reported being confused about the care given or not taking appropriate consent before initiating treatment. For example an adult mother was not happy when her baby was kept in the same incubator with another child: *They put another baby in the same glass house (incubator) as mine… was that right? Different children have different illnesses, other beds (incubators) were empty” (IDI09 Adult mother of a preterm baby).* Similarly, a grandmother left the hospital with the baby when the service providers initiated treatment without asking: *“The grandmother started arguing why the baby was given the medicine. She removed all the equipment, washed the baby’s head, and left next morning…” (IDI09 Adult mother of a preterm baby).* Surprisingly, another mother of a preterm baby also mentioned how they were forced to move from one health facility to another, causing delays, based on the assumption that wouldn’t be able to pay for the treatment as the excerpt below reveals:

*“They (private hospital) said my baby is too small and the costs are too high here. We said we will pay, but they said they don’t want to take the risk and asked us to take baby to the government hospital.” (IDI33 Adult mother of a preterm baby)*.

## Discussion

The findings from this study contribute to our understanding of newborn health and care of preterm babies among mothers including adolescents in rural Baliakandi from the perspectives of the parents themselves, as well as family and community members, and health care providers. The findings have been categorized into two major areas of exploration: (i) perceptions and understanding of PTB and (ii) care practices and care-seeking for preterm babies. In general, findings highlight poor understanding of PTB, with major gaps in care and care-seeking practices among all, including adolescents. We observed gaps and variations in understanding of preterm birth (length of gestation, appearance, causes, problems faced) and care practices (thermal management, feeding, weight monitoring) among all, but particularly among adolescents. Adolescents were found to be largely dependent on family members for these care practices for their preterm babies. The use of multiple providers and delays in care-seeking from trained qualified providers for sick preterm babies was noted. Factors affecting appropriate care seeking included perception of severity of illness, cost, convenience, and quality of services. Health systems challenges included lack of equipment, supplies and trained staff in facilities to provide special care to preterm babies. This findings can enable public health practitioners and policy makers to design the health systems’ response to PTB in a locally relevant way.

The findings revealed variations and gaps in knowledge among the participants about various pregnancy issues including normal pregnancy duration, and perceived causes and consequences of PTB. The perceptions regarding normal pregnancy duration varied between nine months and 10 months and 10 days. A baby born in the 7th or 8th month was considered preterm. This finding is similar to reports from Malawi, Ghana and Uganda, where gestational age at the community level was quantified in months with babies born before nine months being considered to be preterm [21–24, 27]. Accurately estimating the gestational age is crucial for facilitating care-seeking for PTB and the provision of life saving and time-sensitive interventions. The prevalence and preference for home births (51%) in Bangladesh is still high, and unless mothers are educated about PTB and the risks it poses, care-seeking and facility birth even for preterm labor will continue to be low [18, 28]. In addition, similar to other studies in Bangladesh, our findings also reveal a high acceptance and usage of ultrasonography (80% in 2017). However USG is mostly utilized in the latter trimesters when its accuracy for estimating the due date of delivery is less than in the first trimester even though it may still provide some information about fetal growth and wellbeing [18]. Therefore, educating mothers about the value of early pregnancy USG will improve the accuracy of gestational age estimation and consequently the diagnostic accuracy of premature labor and birth. This would ultimately lead to more prompt care-seeking for threatened preterm labor, leading to improved risk mitigation by interventions such as antenatal corticosteroid and magnesium sulphate therapy to reduce the risk of respiratory distress syndrome and neurological sequelae respectively.

Most of the mothers (adults and adolescents) in this study had limited knowledge of the causes of PTB. Those who demonstrated some awareness volunteered a mix of bio-medical and supernatural/spiritual explanations and practices for PTB. The commonly perceived causes e.g., heavy work/stress, inadequate nutrition/food intake, trauma to the abdomen etc., were in line with qualitative studies conducted in similar resource poor settings [22, 23, 29]. While this is encouraging, participants either failed to identify some potential associations with PTB or held beliefs that could deter appropriate antenatal or newborn health care-seeking. For example, contrary to the studies in Malawi, young maternal age was highlighted as a risk factor only by a few fathers in IDIs and FGDs [22, 24, 29]. Participants did not appreciate that previous history of miscarriage or PTB, and multiple pregnancy were risk factors for PTB, often attributing it to supernatural causes such as evils spirits or God’s will [22, 24, 29]. This is consistent with the findings from previous studies in rural Bangladesh and elsewhere, all of which deter women from antenatal care-seeking and encourage resort to spiritual treatments from religious leaders and faith-healers and traditional practitioners [25, 30–32]. The misperception of the mothers regarding PTB requires concerted efforts at culturally congruent education to improve care seeking in their future pregnancies. Indeed, Legare et al. (2012), highlights the pivotal role of understanding the extent to which traditional perinatal explanations and practises compete, conflict and coexist to provide unique insight into cultural ecologies of health, and asserts that this is critical to improving the efficacy of health education interventions and policies [33, 34]. In addition, the social capital associated with traditional medicine could also be capitalised to improve knowledge of the community regarding PTB and appropriate referral for high-risk preterm babies.

In general, breastfeeding improves neurodevelopmental outcomes and protects preterm babies from sepsis, necrotizing enterocolitis, and retinopathy of prematurity [35–38]. Breastfeeding was highly valued by the community in our study, primarily, as a means to improve the health of the weak and under-nourished preterm babies. Despite this, we also observed non-recommended practices such as prelacteal feeding, and non-exclusive breastfeeding. Several studies exploring care in newborn or low-birth-weight newborns in Bangladesh and similar settings also report that newborns or babies are primarily supplemented with either formula milk, cow or goats’ milk or porridge (rice, sugar and water) when the mothers feel that they are not producing enough milk or are insufficiently lactating, whereas prelacteal feeds are usually introduced as a ritual practice, “to help keep baby’s throat and stomach clear”, or due to a delay in the flow of breastmilk [39–43]. Often, the suggestion of the formula feed is made upon consultation with formal or informal care providers [43]. Overall, in Bangladesh, 87% of women who experienced facility births were counseled on exclusive breastfeeding. This has resulted in a decrease in the proportion of children who are given prelacteal feed and an increase in non-exclusive breastfeeding [18]. Even so, about 29% of children under-2 years are given prelacteals and 35% of children 0–5 months are not exclusively breastfed, according to the 2017-18 BDHS report [18]. This highlights that further improvements can be made, especially among young mothers or mothers of preterm babies, for example by training neonatal nurses or health workers to provide home or facility-based evidence-informed breastfeeding support [44–46].

The community in Baliakandi did not recognize hypothermia to be a separate entity but were conscious that newborns, and specifically preterm babies, are at high risk of “catching cold” and indicated preventative practices such as bathing babies at noon with warm water, the use of emollients, and appropriate dressing of babies according to the external temperature [31, 42, 43, 47]. None of the participants, including those who gave birth in facilities, was aware of or had practiced KMC. The community still valued early bathing, but bathing was more likely to be delayed by three or more days for neonates born at health facilities or upon advice of health care providers. While these practices do not fully protect newborns from hypothermia, it is evident that the community in Baliakandi as well as those in Sylhet, Gopalgonj [31, 40, 42], placed a high value on keeping babies warm. Efforts to implement and improve the implementation and adoption of appropriate thermal care and KMC for preterm babies can capitalize on this pre-existing awareness [31, 40, 42]. However, KMC has been scaled up in only 314 facilities in Bangladesh, with less than 5% of preterm newborns receiving KMC in 2021 [48]. Our findings highlight the need for further concerted effort to accelerate training and scale up of KMC in rural communities and facilities by addressing health systems challenges and demand-side barriers.

Although programming often targets interventions to preterm babies, the emergent themes in this study suggest that the local explanatory model of PTB does not always consider being preterm as a determinant or stimulus for health care-seeking. Instead, community members considered various observable characteristics and illnesses of the baby or newborn to first judge the overall health. According to the participant narratives, it was natural for preterm babies to be small and weak. In the absence of any visible illnesses or symptoms, many of these babies were considered to be healthy babies who would grow well if fed appropriately. As a result, many at-risk babies, especially late PTBs, in this community are overlooked or care-seeking delayed due to the inability of caregivers to identify risk factors, spot danger signs and assess severity. In agreement with studies in Uganda and Malawi babies were considered at high risk and more likely to receive care when they were very small, had fused eyelids, thin or wrinkled skin, or breathing problems etc. [21, 22, 24, 29]. Similarly, studies in Bangladesh and similar settings have associated recognition of symptoms of illness and danger signs with increased care-seeking for sick preterm children. This reiterates the need to educate caregivers on recognition of risk factors for mortality and morbidity [25, 49, 50].

A few prior studies in rural Bangladesh and other low-resource settings also highlighted perception of inevitability or fatalism in preterm or sick newborns as a deterrent for care seeking [51–53]. Although, participants in our study repeatedly mentioned that babies born as early as seven months may not survive, their views were not fatalistic but rather the overall opinion was to seek care from qualified/formal health care providers for such babies. Several participants also mentioned the use of incubators or challenges of having to spend a lot of money for formal care, indicating a shift in beliefs towards acknowledging that extremely preterm babies can also survive.

The local explanatory model for newborn illness in Baliakandi, as in other parts of Bangladesh, or other low-resource settings is largely influenced by a wider ecological framework including the management options available and other factors related to the local health system. In Baliakandi, participants reported several barriers to accessing formal health care - financial constraints, quality issues (lack of services, trained staff and equipment, inadequate referral system etc.) and disrespectful care. They demonstrated a preference for informal care by unqualified providers, consistent with prior studies that have highlighted that only a small proportion of families sought care from formal providers [25, 51, 54, 55]. Equally, there is ample evidence regarding the limited availability of good quality care at health facilities including in Bangladesh [21, 22, 56–58]. Care-seeking decisions regarding newborns and children is often influenced by family members, relatives, neighbors and peers whose narratives often reinforce reports of mistreatment in facilities in contradistinction to positive views regarding traditional care [58, 59]. Improving access to and quality of preterm and newborn care at health facilities, delivered respectfully, is therefore an imperative to drive shifts in the care-seeking behavior and subsequently the local explanatory model [58, 60].

Our study also revealed that most adolescents lived with their extended families and were highly dependent on their social network particularly natal and marital kins for support [32, 41]. Instrumental and informational support for care of their baby was provided primarily by their mother and mother-in-law, whereas financial support was provided by their husband and other family members. As such, decision making related to care and care-seeking for sick babies was never made in isolation but rather jointly taken with husbands and family members. In rural Bangladesh where adolescents lack knowledge and decision-making authority, health education on preventative practices and management practices of the preterm babies should, therefore, be provided at the family level involving women, their husbands, and the mother/mothers-in-laws [32, 41].

One of the strengths of this study is the inclusion of different methods and participants to triangulate the findings. We conducted IDIs, FGDs and KII with participants from different ages (adults and adolescents), birth status (preterm, term), genders, and relationship with the babies, allowing us to report similarities and differences in values and beliefs. Despite the benefits, summarising perspectives of multiple groups of participants and reporting their findings was challenging. In order to ensure that we do not over emphasise or marginalise any group and to refine our interpretations, we conducted a thematic analysis revisiting the data and summaries, and conducting regular discussions with the team involved in data collection and analysis to enhance the depth of the summary. Participants with preterm and term births were identified from the existing surveillance system and birth status (preterm, term) were also verified during interviews. However, data was collected retrospectively and may be subject to recall bias. To minimize recall bias we included participants within 6 months of birth.

## Conclusion

A combination of factors including local knowledge, socio-cultural practices and health systems challenges influenced awareness of, and care for, preterm babies among adolescent and adult mothers and the community. Strategies to improve birth outcomes will require increased awareness among adolescents, women and their families about PTB and improvement in the quality of, and access to, PTB services at health facilities – especially for adolescent mothers. In addition, intervention strategies to improve care of preterm babies should account for social, cultural, and economic reasons for current practices. Additionally, improved quality and competence of health facilities in managing care for preterm babies is likely to motivate appropriate care-seeking and improve chances of survival for those who do seek care.

## Data Availability

The datasets used and/or analysed for the current study are available from the corresponding author on reasonable request.

## Abbreviations

BDHS: Bangladesh Demographic and Health Survey
FGD: Focus group discussion
IDI: In-depth interview
KII: Key informant interview
LMIC: Low- and middle-income country
PTB: Preterm birth
USG: Ultrasonography
